# A Medical Conundrum in a Surgical Setting: Lessons Learned From an Atypical Case of Hyponatremia

**DOI:** 10.7759/cureus.31061

**Published:** 2022-11-03

**Authors:** Ashutosh Kapoor, Fatima Senra, Ian Stone, Mahesh M Deore

**Affiliations:** 1 Diabetes and Endocrinology, Imperial College Healthcare NHS Trust, London, GBR; 2 Surgery, Northwick Park Hospital, London, GBR; 3 Respiratory Medicine, Northwick Park Hospital, London, GBR; 4 Diabetes and Endocrinology, Northwick Park Hospital, London, GBR

**Keywords:** rare genetic diseases, rare cause of acute abdominal pain, porphyria, syndrome of inappropriate antidiuretic hormone secretion, hyponatremia

## Abstract

In the world of medicine and specifically endocrinology, hyponatremia is one of the commonest electrolyte abnormalities that result in a varied spectrum of presentations. Patients can incur symptoms ranging from lethargy, light-headedness, and confusion to much more severe symptoms such as vomiting, abdominal pain, deterioration in consciousness, and, in critical cases, even seizures. In elderly patients, hyponatremia is a major cause of Delirium and if not treated appropriately, can result in adverse outcomes and complications.

In severe cases of hyponatremia, the opinion of an endocrinologist must be sought early for a conscientious investigation of the underlying etiology, as this prevents the need for unnecessary interventions, and thus reduces the risk of potential harm. Despite being a common electrolyte abnormality, hyponatremia can be associated with rare and uncommon etiologies, one of them being acute intermittent porphyria (AIP) as seen in our case. Due to the non-specific presentation of AIP, medical and healthcare professionals must be cautious of this condition, since it can mimic an acute abdomen. Symptoms of AIP usually overlap with other conditions, thus resulting in a diagnostic dilemma.

Triggers and factors leading to acute attacks of AIP must be explored and rationalized appropriately, involving a thorough review of a patient’s medication and social history. Moreover, discussion in a multidisciplinary team (MDT) setting for such complex presentations has a positive impact on patient care and is therefore recommended.

## Introduction

Electrolyte abnormalities are commonly seen in both hospitalized patients and those presenting to the Emergency Department (ED), and among the commonly encountered electrolyte abnormalities are hyponatremia, hypokalemia, hyperkalemia, and disorders of calcium and magnesium [1].

Hyponatremia is one of the commonest electrolyte abnormalities encountered, and it is usually caused by an imbalance in total body water and sodium content; namely, solvent and solute. On the basis of fluid status, hyponatremia can be subdivided into hypovolemic hyponatremia, euvolemic hyponatremia and hypervolemic hyponatremia [2]. Thus, early fluid status assessment of a patient with hyponatremia is key in the formulation of the subsequent management plan. We recommend that in cases of severe hyponatremia, the opinion of an endocrinologist be sought early for appropriate investigations and prompt formulation of an optimal management plan. Furthermore, exhaustive efforts must be made to unearth the underlying etiology of hyponatremia. 

Despite being a common electrolyte abnormality, hyponatremia can be associated with rare and uncommon etiologies, one of them being acute intermittent porphyria (AIP) as depicted in our case. This case report focuses on the importance of thinking out of the box wherein common conditions and causes of hyponatremia have been ruled out, illustrating the importance of considering rarer medical causes of the “acute abdomen” where surgical causes have been excluded, following extensive investigations.

AIP is a rare autosomal-dominant (AD) metabolic disorder characterized by a deficiency of the enzyme Porphobilinogen Deaminase (PBGD), and if left undiagnosed, can result in recurrent unnecessary investigations and potential for harm. AIP is characterized by incomplete penetrance ranging from 10 to 20% and clinically, this subset of AIP carriers tends to develop acute attacks and symptoms, thus categorized as manifest AIP [3]. Hyponatremia is the commonest electrolyte manifestation in the cohort of patients with a background of AIP [4], and the underlying mechanism usually causing hyponatremia involves a syndrome of inappropriate antidiuretic hormone secretion (SIADH) [5,6], although renal salt wasting and intestinal losses of sodium have also been documented.

AIP is usually prevalent and manifests in women in their second through fourth decades of life, and the symptoms are predominantly comprised of sympathetic overdrive, recurrent bouts of abdominal pain as well as neurologic symptoms such as confusion, depression, and altered mental status [7]. Due to inborn errors in metabolism, there is an accumulation of by-products such as porphobilinogen (PBG) and aminolevulinic acid (ALA) in AIP [7].

This article was previously presented as an oral communication at the National Clinical Cases Conference, 2022 conducted by the Society for Endocrinology on June 15, 2022 at the Royal Society of Medicine, London.

## Case presentation

First admission

We report the case of a 25-year-old South-Asian male who was admitted to our hospital with acute abdominal pain and hyponatremia, following recent emigration to the United Kingdom (UK). The episodes of abdominal pain spanned over a duration of four years; however, over the course of the last two months, the frequency and intensity had considerably worsened. His previous surgical history was significant for laparoscopic appendicectomy, following which he underwent a laparotomy and adhesiolysis for postoperative small bowel obstruction (SBO) as a sequela of postoperative complications in his home country in 2019.

Furthermore, he also recollected having some trouble with “salts in his body” in the past as informed by the medical personnel in his home country. He denied significant alcohol intake or use of illicit drugs and was a non-smoker. He did not take any regular or over-the-counter (OTC) medications. He was admitted to the surgical team due to a high index of suspicion of surgical pathology and clinical signs in keeping with an acute abdomen.

**Figure 1:**
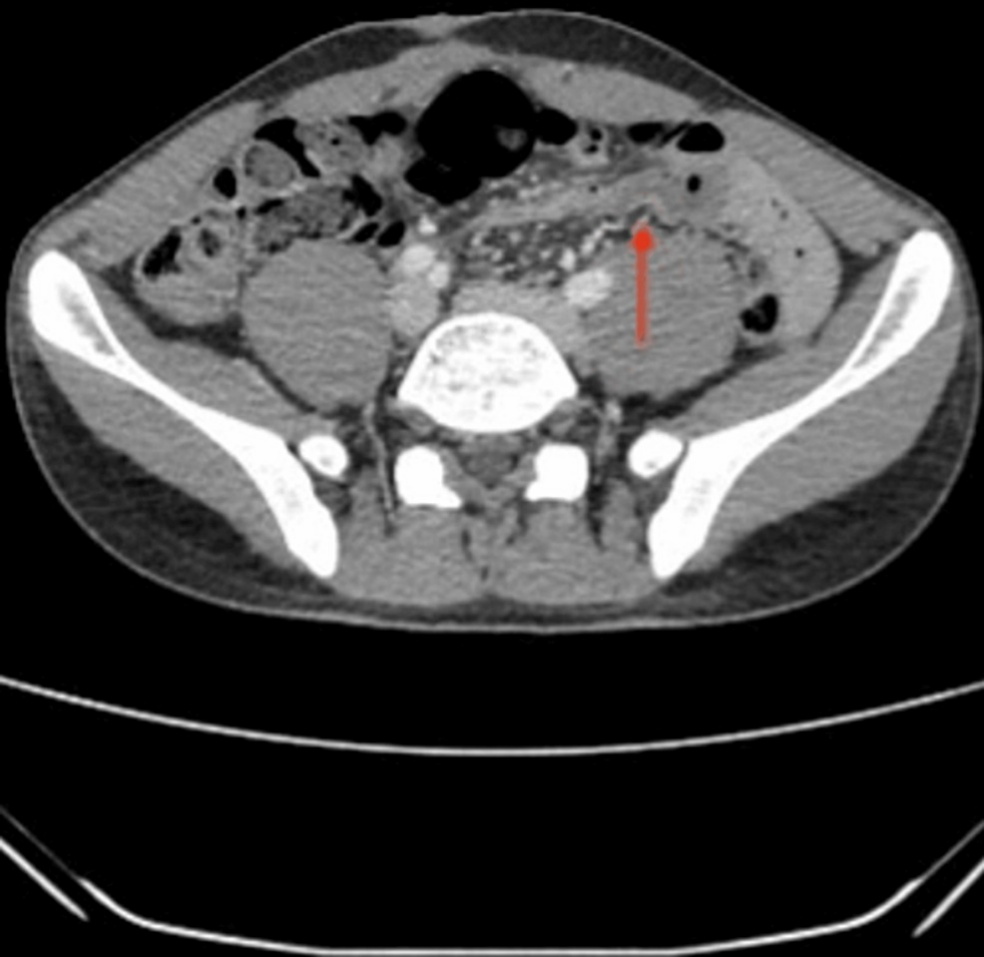
There is no evidence to support a high-grade obstruction or ischemic bowel. The described apparent abrupt caliber change within the distal ileum is nonspecific but could represent subacute or intermittent small bowel obstruction (SBO), particularly given the patient's past surgical history. Arrow shows transition point in terminal ileum.

Computerized tomography (CT) scan of the abdomen and pelvis was conducted during this admission for the assessment of surgical pathology (Figure 1). This revealed no evidence of a high-grade obstruction of the ischemic bowel. Due to the non-specific nature of findings in the distal ileum, there remained a radiological suspicion of subacute or intermittent SBO.

Initial management and treatment were via a conservative approach with Gastrograffin and naso-gastric tube (NGT) decompression; however, despite these measures, since the symptoms did not resolve, a decision was made to proceed with surgical intervention. An exploratory laparotomy and adhesiolysis were performed as a closed-loop SBO at the terminal ileum caused by adhesions from the previous surgeries were found.

Unfortunately, during the post-operative recovery phase, the patient deteriorated clinically whilst on the surgical ward, which was reflected by a decline in GCS from a baseline of 15/15 to 13/15. He was reviewed by the intensive treatment Unit (ITU) team and found to have severe hyponatremia of 103 mmol/L (Normal levels: 135-145 mmol/L). On admission, the initial sodium level was 120 mmol/L. This was managed at the time via a conservative approach and cautious biochemical monitoring. Prior to surgery, the patient had stable Sodium levels and did not develop any new symptoms of clinical concern. Due to the acute drop in Sodium levels, accompanied by clinical deterioration, a decision was made to proceed with the administration of 250 mL of 1.8% strength hypertonic saline, following which he was admitted to ITU for further management and strict fluid status monitoring. A repeat sodium assay conducted after the administration of hypertonic saline was 108 mmol/L. There was a noticeable improvement in his clinical picture and consciousness level following this. CT Head conducted prior to the administration of Hypertonic Saline ruled out any intracranial pathology, and the deterioration in GCS was attributed to the decline in Sodium levels. He developed no new focal neurology or signs after the above-mentioned treatment. Subsequent magnetic resonance imaging (MRI) brain, which was conducted for completion, did not show any evidence of pontine myelinolysis.

Specialist input was sought, and a management plan was formulated by the Endocrinology team. This included a thorough and extensive workup for hyponatremia as seen in Table 1. Blood glucose and ketones levels were within normal limits.

**Table 1:** Investigations for Hyponatremia

Investigations	Results	Reference range
Thyroid Stimulating Hormone	1.87 mIU/L	0.27-4.20 mIU/L
Cortisol (09:30 am)	491 nmol/L	110-550 nmol/L
Lipid profile	Cholesterol 4.7 mmol/L	2.3-4.9 mmol/L
	Triglycerides- 1.2 mmol/L	0-2.2 mmol/L
Serum Osmolality	233 mmol/kg	275-295 mmol/kg
Urine Osmolality	377 mOsm/kg	50-1200 mOsm/kg
Urinary Sodium	135mmol/L	<20mmol/L

Given that the patient was euvolemic, in conjunction with an elevated Urinary Sodium with alternative causes having been ruled out, SIADH was diagnosed. Fluid restriction was commenced and intensified to 750 milliliters/day due to the lack of response to the initial fluid restriction of 1.5 L, with daily reviews by the Endocrine team. These measures resulted in a brief period of improvement in sodium levels; however, the levels started to deteriorate once again. Therefore, oral Urea was commenced as per the hospital protocol at a starting dose of 15 gm/day and up-titrated to 60 g/day to promote free water excretion. This led to a gradual improvement in both, biochemical and clinical parameters. Nevertheless, despite evaluation with radiological imaging including chest x-ray and CT imaging of the head, abdomen, and pelvis, the cause of SIADH remained unknown. The patient was safely discharged with a follow-up plan in place. However, he was unfortunately lost to follow-up due to non-compliance with both the outpatient appointments that were organized.

Second admission

**Figure 2:**
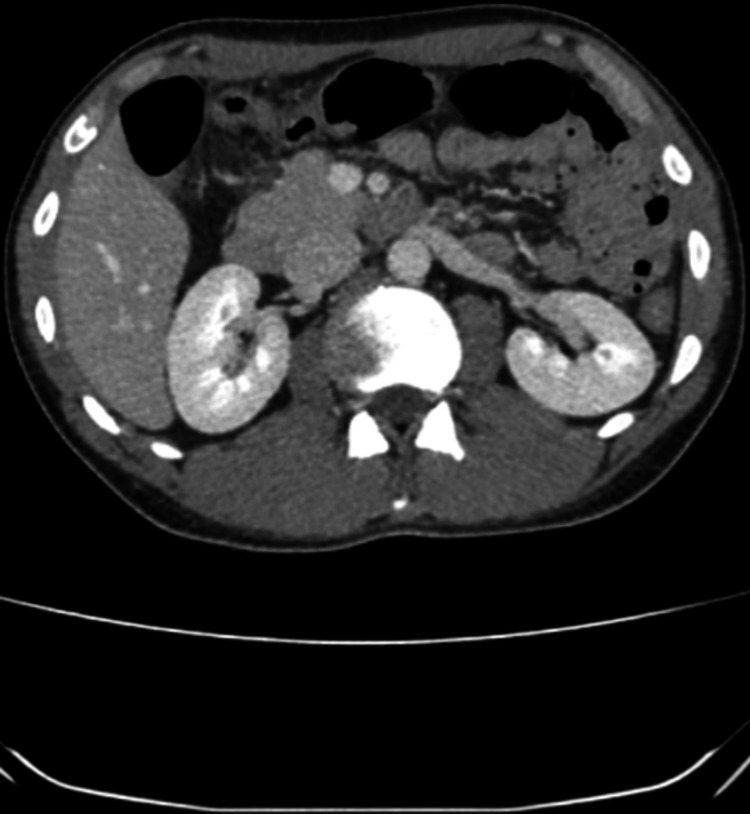
Stranding in the anterior abdominal wall in keeping with previous laparotomies. Normal appearances of the unprepared small and large bowels. No free fluid in the abdomen and pelvis. Normal appearances of the lung bases, adrenal, pancreas, liver, gall bladder, and kidneys. No cause was identified on CT imaging for the patient’s symptoms.

Three months following initial discharge, the patient was re-presented to our hospital’s ED in March 2022, with similar symptoms. On this occasion, he was admitted directly under the surgeons and the urgent input of an endocrinologist was sought on account of the initial complex admission and hyponatremia of 118 mmol/L (normal: 135-145 mmol/L), on current up-to-date blood. A repeat CT abdomen and pelvis (Figure 2) conducted due to suspicion of acute abdomen is as below. There was no discernible cause identified on imaging that could explain the patient's symptoms, with normal appearances of the small and large bowels.

To ensure strict electrolyte and vigilant clinical monitoring, he was transferred to the Medical High Dependency Unit (mHDU), where his stay was complicated by recurrent episodes of confusion and severe hypertension consistent with sympathetic overactivity. The National Early Warning Score (NEWS) charting was reflective of episodic hypertension, predominantly the diastolic component. The highest documented blood pressure (BP) was 180/120 mmHg, with hypertensive spells documented during the episodes of abdominal pain. The appearances of the Adrenal glands on the CT Abdomen and Pelvis, as seen above, were unremarkable, thus making an Adrenal-related pathology unlikely. A CT Head conducted during this admission was reported to be normal with no evidence of any intracranial pathology.

Sodium levels improved once again with the implementation of fluid restriction and the usage of urea tablets. Despite gradual improvement and eventual normalization of sodium levels, he continues to experience episodes of hypertension and abdominal pain. Due to the lack of any surgical pathology following extensive investigations, a porphyria screen was requested as one of the rare medical causes of abdominal pain and severe hyponatremia. Preliminary local lab results revealed significantly elevated random PBG and total porphyrin levels in urine. PBG levels were elevated at 310 micromoles/L (normal: 0-90 micromoles/L).

For specific testing, investigations including urine and feces samples were sent to the regional tertiary porphyria center to confirm the provisional diagnosis. The results are given in Table 2.

**Table 2:** Porphyrins screen

Investigations	Results	Reference range
5-aminolevulinic acid (ALA)	45.5 µmol/mmol creatinine	<3.8
Porphobilinogen (PBG)	56.1 µmol/mmol creatinine	<1.5
Total porphyrins	663 nmol/mmol creatinine	<35
Total uroporphyrin (I + III)	6,359 nmol/L	<24
Hepatcarboxylate porphyrin	181 nmol/L	<4
Total coproporphyrin (I + III)	748 nmol/L	<115

The above results were confirmatory of the diagnosis of Porphyria, following which the patient was discussed and referred to the specialist Porphyria team for further management and consideration of Heme products. It was recommended by the specialist Porphyria team to administer Heme products during the acute episode, in order to shorten the duration and alleviate the intensity of the episodes. Post discharge, he has remained well whilst off Urea tablets and has not warranted any further admissions. Clinically, there have been no new acute issues and has an ongoing follow-up with both the Endocrinology and Porphyria teams.

## Discussion

Hyponatremia is a common electrolyte abnormality, which can be associated with uncommon etiologies as noted in our case; it is thus prudent to adopt a careful and methodical approach to assessing the underlying etiology. Suarez et al. reported a case of hyponatremia in the context of AIP where the histopathological examination revealed a marked decrease in the number of hypothalamic cells. Thus, it could be hypothesized that hypothalamic-hypophyseal tracts may be damaged in this cohort of patients, leading to the development of SIADH [6,7].

AIP is one of several disorders that arise from enzyme-related derangements in the biosynthetic pathway of the Heme molecule. The enzyme deficiency that usually occurs is that of hydroxymethylbilane synthase (HMBS), also known as PBGD [7]. AIP is the most common of acute porphyrias across the globe, with an estimated prevalence of approximately five per 100,000 people, and due to its autosomal dominant pattern of inheritance, patients with this condition usually have an associated and known family history. However, there was no known family history in our case. AIP being an autosomal dominant condition with a low penetrance means that majority of these patients may remain asymptomatic. It is estimated that the penetrance of symptomatic disease is approximately 1% of all AIP gene carriers [8].

When no surgical causes are found to explain abdominal pain following extensive investigations as seen in our case, rare medical causes must be explored. Symptoms of AIP usually have a gradual progression that develops over the course of several days and weeks, and during the acute attack, abdominal pain occurs due to impaired intestinal motility related to autonomic nerve dysfunction [8]. Systemic hypertension is also a feature of autonomic neuropathy in acute attacks.

Being a male patient, our case contrasts the usual gender prevalence of symptomatic AIP, which is more commonly seen in females, i.e., approximately 90% of symptomatic patients are female, usually women of reproductive age, with symptomatic bouts typically commencing after the onset of menses [8]. In women with AIP, attacks are generally exacerbated during the luteal phase of the menstrual cycle, although in many patients, there is no obvious pattern or trigger. Usual precipitants comprise factors such as alcohol intake, smoking, use of illicit drugs, and intercurrent illness or infection. Change in urine color, especially on light exposure, is often a pathognomonic feature of AIP and occurs due to the oxidation of PBG to uroporphyrin and Porphobilin [8], although there was no documented urine discoloration in our patient.

In the literature, there have been various theories that have been documented that may cause hyponatremia. Another proposed theory is that excess PBG and ALA lead to development of SIADH via the toxic effect being exerted on the neurological system. This effect stimulates the paraventricular nucleus in the hypothalamus, causing excessive ADH secretion, thus resulting in hyponatremia [9].

This case highlights the importance of considering the diagnosis of AIP in patients presenting with recurrent abdominal pain and hyponatremia after excluding surgical causes. Due to its complex and non-specific symptomatology, AIP is often misdiagnosed as a result of the diagnostic conundrum and is thus, missed, which also increases the likelihood of carrying out unnecessary investigations. Therefore, in the UK, the National Acute Porphyria Service provides clinical advice and treatment options when indicated.

## Conclusions

SIADH is a diagnosis of exclusion and should be considered where all other potential causes for hyponatremia have been assessed and excluded, and once confirmed, the next step is to investigate for the underlying etiology, since treating the instigating cause results in improvement and resolution of hyponatremia.

We recommend that in cases of severe hyponatremia, the opinion of an endocrinologist be sought early for appropriate investigations and prompt formulation of an optimal management plan. A conscientious effort must be made to discover the underlying etiology of hyponatremia, as this would prevent the need for interventions that are not warranted, thus reducing the risk of potential harm and the burden of unnecessary investigations.

## References

[1] Tazmini K, Nymo SH, Louch WE, Ranhoff AH, Øie E (2019). Electrolyte imbalances in an unselected population in an emergency department: a retrospective cohort study. PLoS One.

[2] Sahay M, Sahay R (2014). Hyponatremia: a practical approach. Indian J Endocrinol Metab.

[3] Puy H, Gouya L, Deybach JC (2010). Porphyrias. Lancet.

[4] Yang J, Chen Q, Yang H (2016). Clinical and laboratory features of acute porphyria: a study of 36 subjects in a Chinese tertiary referral center. Biomed Res Int.

[5] Meersseman W, Cassiman D, Goossens W, Vanderschueren S (2008). An unusual cause of syndrome of inappropriate antidiuretic hormone secretion. Acta Clin Belg.

[6] Suarez JI, Cohen ML, Larkin J, Kernich CA, Hricik DE, Daroff RB (1997). Acute intermittent porphyria: clinicopathologic correlation. Report of a case and review of the literature. Neurology.

[7] Wang B (2021). The acute hepatic porphyrias. Transl Gastroenterol Hepatol.

[8] Sassa S (2006). Modern diagnosis and management of the porphyrias. Br J Haematol.

[9] Solares I, Tejedor M, Jericó D, Morales-Conejo M, Enríquez de Salamanca R, Fontanellas A, Tejedor-Jorge A (2020). Management of hyponatremia associated with acute porphyria-proposal for the use of tolvaptan. Ann Transl Med.

